# Recent Progress in Heteroatom-Containing Metalloporphyrin-Based Catalysts for CO_2_ Reduction

**DOI:** 10.3390/molecules30112287

**Published:** 2025-05-23

**Authors:** Zhuo Li, Qianqian Wei, Zhixin Ren, Jing Xie

**Affiliations:** 1 Key Laboratory of Cluster Science of Ministry of Education, Beijing Key Laboratory of Photoelectronic/Electrophotonic Conversion Materials, School of Chemistry and Chemical Engineering, Beijing Institute of Technology, Beijing 100081, China; zhuo@bit.edu.cn (Z.L.); weiqianqian@bit.edu.cn (Q.W.); 2 School of Chemistry, Chemical Engineering and Materials, Jining University, Qufu 273155, China

**Keywords:** metalloporphyrins, density functional theory calculation, catalytic performance, coordination environment, mechanisms

## Abstract

Metalloporphyrins, owing to their structural resemblance to natural enzyme active sites and highly tunable coordination environments, have emerged as promising catalysts for converting CO_2_ into value-added chemicals and fuels. Considerable efforts have been made to modify metalloporphyrins to improve their catalytic capability for CO_2_ reduction. One approach involves modifying the metal coordination environment (known as the first coordination sphere) to generate heteroatom-containing metalloporphyrins, particularly N-confused and O/S-substituted variants. While heteroatom-containing metalloporphyrins were first synthesized in 1989, their use in CO_2_ reduction catalysis was not reported until after 2020. Herein, we review the recent progress in the design, catalytic performance, and mechanistic studies of N-confused and O/S-substituted metalloporphyrins towards CO_2_ reduction. This review encompasses both experimental and theoretical computational work, as well as the use of porphyrins as catalysts in photocatalysis and electrocatalysis. Finally, based on the current research advances, we present critical recommendations and future research directions, with a focus on theoretical studies, in the hope of facilitating the rational design of novel catalysts for sustainable energy conversion and environmental remediation.

## 1. Introduction

The increasing concerns about climate change and the depletion of fossil fuels have raised the need for sustainable energy solutions [1,2]. The electrochemical reduction of carbon dioxide (CO_2_) to value-added hydrocarbons and chemical feedstocks, such as alcohols, aldehydes, ketones, etc., represents a promising approach for dealing with CO_2_ emissions and alleviating energy and environmental crisis [3,4,5]. However, the inherent chemical inertness of CO_2_ and the existence of a competitive hydrogen evolution reaction poses a challenge for its efficient conversion [6,7,8], thereby driving the need for catalyst development [9,10].

Metalloporphyrins are a class of coordination complexes in which a metal ion (e.g., Fe, Mg, Co, Zn, Cu, Ni) is bound to the nitrogen atoms of a porphyrin ligand. They are ubiquitous in nature and serve essential functions in various critical life processes. Typical examples include iron porphyrins (e.g., heme and cytochrome P450), magnesium porphyrins (e.g., chlorophyll), and cobalt porphyrins (e.g., vitamin B12) [11,12]. These metalloporphyrins share a common mechanistic hallmark: enabling electron transfer via redox transitions of the central metal ion (e.g., Fe^2+^/Fe^3+^) or activating small molecules (e.g., O_2_, CO_2_) through metal–ligand coordination [13,14]. Their unparalleled catalytic versatility has driven breakthroughs in artificial enzyme design, with transformative applications in precision medicine like targeted drug metabolism and renewable energy, particularly CO_2_-to-fuel conversion [15,16,17]. Regarding the latter, much empirical evidence has been reported in the literature. For example, iron porphyrin compounds can act as homogeneous catalysts for the reduction of CO_2_ to CO in N, N-dimethylformamide (DMF) solution [18]. Nickel porphyrin compounds featuring exocyclic 1,3-di*tert*-butylimidazolin-2-ylidenamino (NI*t*Bu) substituents also exhibit catalytic activity for CO_2_-to-CO conversion in DMF [19]. Additionally, cobalt porphyrin compounds can be incorporated into metal–organic frameworks (MOFs) or covalent–organic frameworks (COFs) through organic linkers, serving as efficient catalysts for CO_2_ reduction to CO [20,21,22,23,24,25].

**Figure 1:**
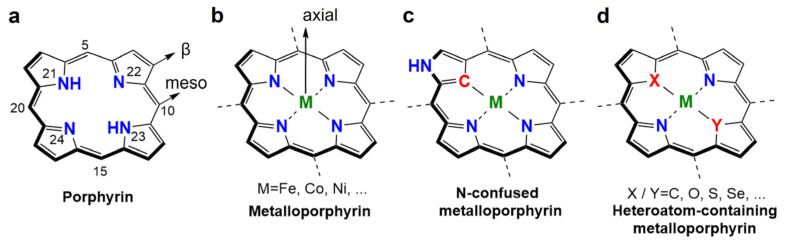
Representative structures of (**a**) porphyrin, (**b**) metalloporphyrin complexes, (**c**) N-confused metalloporphyrin complexes and (**d**) heteroatom-containing metalloporphyrin complexes.

How to optimize the catalytic activity and selectivity of the metalloporphyrin-based catalysts is the key to these studies. To reach this end, modifying the structures of the metalloporphyrins has been shown to be an effective strategy [26,27]. The distinctive structure of porphyrin molecules provides a highly modifiable platform for functionalization. As shown in Figure 1a, porphyrins are macrocycles that are composed of four pyrrole subunits interconnected via methine bridges, forming a planar, aromatic, and highly stable framework [28]. It readily anchors metal ions at its core, forming a stable M–N_4_ coordination environment (Figure 1b) [29]. In addition to changing the type of the central metal, common strategies for modifying metal porphyrins include introducing substituents at the meso or β positions of the porphyrin, coordinating axial ligands to the central metal, and modifying the first coordination or the outer coordination sphere [27,30,31,32,33,34]. Among these strategies, modifying the first coordination sphere of metal porphyrin compounds has received relatively less attention, likely due to the greater synthetic challenges in constructing asymmetrically coordinated metal porphyrin systems. Nevertheless, recent experimental and theoretical studies have made significant progress in this direction, demonstrating it as an effective strategy for enhancing catalytic efficiency [27,35,36,37]. Of note, this strategy has been widely used in graphene-based M–N–C type single-atom catalysts [38,39,40,41,42].

A number of recent review articles have provided comprehensive coverage of utilizing metal porphyrin-based catalysts for CO_2_ conversion [14,26,43,44,45]. These reviews all focus on traditional N_4_-metalloporphyrin systems. For example, Philipp Gotico et al. [44] (2020) and Domingo-Tafalla et al. [26] (2023) reviewed research advances in outer coordination sphere modifications through functional group effects (e.g., hydrogen bonding, electrostatic interactions, and electronic effects) and hierarchical material design (e.g., MOFs, COFs, and carbon composites) of metalloporphyrins. To our knowledge, no review has addressed modifications of the first coordination sphere in metal porphyrins for CO_2_ reduction, motivating our summary of recent advances in this field.

Modifying the first coordination sphere in metal porphyrins enables access to diverse derivatives with potentially improved catalytic performance. For example, N-confused porphyrins, which are isomers of porphyrins, are formed by inverting the N and C atoms of one or multiple pyrrole rings (Figure 1c). Additionally, replacing one or two pyrrole N atoms with other heteroatoms such as O, S, Se, B, P, and C results in a class of porphyrinoids known as heteroatom-containing porphyrins or core-modified porphyrins (Figure 1d) [46,47,48]. Among the latter, metalloporphyrins incorporating oxygen or sulfur heteroatoms demonstrate relatively facile synthesis. These substitutions induce significant modifications in both the geometric and electronic structures of the metalloporphyrins, and they fundamentally change the reaction coordinate and introduce additional mechanistic complexity in CO_2_ reduction catalytic pathways.

Herein, we present a review of the advancements of first coordination sphere modification on metalloporphyrins for CO_2_ reduction, both experimentally and theoretically. We particularly focus on the N-confused and O- or S-substituted species. The contents of this review are organized as follows: Session 2 briefly reviews the application of metalloporphyrins in CO_2_ reduction and introduces the commonly used computational methods in modeling this process. Session 3 reviews the synthesis and catalytic progress of N-confused metalloporphyrin for CO_2_RR. Session 4 reviews the synthesis and catalytic progress of O/S-substituted metalloporphyrin for CO_2_RR. Session 5 presents a summary and outlook. By reviewing the current state of research on first coordination modification on metalloporphyrins, we hope to provide insights into the design principles and strategies that can guide the development of next-generation catalysts for sustainable energy conversion.

## 2. Metalloporphyrins for CO_2_RR

The use of metalloporphyrins as catalysts for CO_2_ reduction can be traced back to 1979. Toshima et al. found that cobalt porphyrins with carboxyl (–COOH) and sulfonic acid groups (–SO_3_H) showed electrocatalytic activity in reducing CO_2_ to formic acid, HCOOH [49]. Since then, metalloporphyrins have garnered increasing attention as catalysts for CO_2_ reduction, especially those with low-cost transition metal centers like iron, cobalt, and nickel [18,19,43,50,51]. For example, Savéant et al. (2016) reported electrochemical CO_2_-to-CO conversion by Fe tetraphenylporphyrins with four positively charged trimethylanilinium groups (Fe-*p*-TMA) [33]. The Omar Yaghi group (2015) synthesized co-porphyrin covalent organic frameworks (COF-366-Co) for the electrocatalytic reduction of CO_2_ to CO [20]. The Koper group (2015) immobilized cobalt porphyrins on pyrolyzed graphene as electrocatalysts to reduce CO_2_ to CO and CH_4_ [52]. Dey’s group (2021) achieved the conversion of CO_2_ to HCOOH via electroreduction by introducing a pendant amine group onto the iron porphyrin (Fe-2,3,7,8-tetracetyl-15-phenylporphyrin, FeTEsP) [53]. Liu et al. (2013) reported copper porphyrin-based metal–organic frameworks (Cu-5,10,15,20-tetrakis(4-carboxyphenyl) porphyrin, Cu-TCPP-MOFs) as photocatalysts for converting CO_2_ to CH_3_OH [54].

The above studies indicate that metal porphyrin compounds predominantly catalyze the reduction of CO_2_ to C1 products, including carbon monoxide (CO) [18,20,31,33,50,55], formic acid (HCOOH) [49,53], methanol (CH_3_OH) [54], and methane (CH_4_) [52,56,57]. C2 products are scarcely observed on porphyrin-based catalysts because of the lack of suitable adsorption sites for C–C coupling to C2 intermediates. Hence, modifications to the coordination sphere of metal porphyrins retain this selectivity, with C1 species—particularly CO—remaining the dominant reduction products.

**Figure 2:**
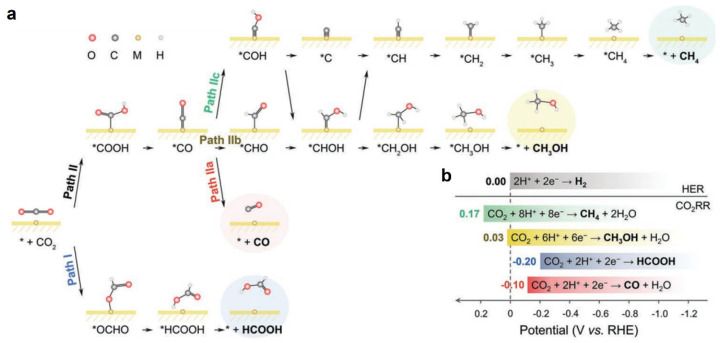
(**a**) Possible CO_2_RR reaction pathways (under CPET assumption) and products with (**b**) corresponding overall reaction equations and standard redox potentials [71].

Elucidating the precise mechanism of CO_2_ reduction by metalloporphyrin compounds faces several fundamental challenges: the complexity of possible reaction pathways sensitive to experimental conditions, the transient nature of key intermediates limiting experimental detection, and ambiguous electronic configurations arising from redox-active metal centers and porphyrin ligands [58,59]. Hence, a computational study plays an important role. In theoretical calculations, density functional theory (DFT) is almost exclusively employed. Researchers have adopted two distinct modeling approaches, molecular cluster-based DFT calculations and periodic DFT calculations. Molecular cluster models are used for molecular metalloporphyrin complexes as homogeneous catalysts. Electron transfer (ET) and proton transfer (PT) processes are typically treated separately [56,60,61,62,63,64,65,66], enabling the computation of reduction potentials for different species and identifying the order and/or synchronicity of ET and PT. The periodic DFT calculations, which are widely used for heterogeneous catalysts, are primarily applied to covalent organic frameworks (COFs) and metal–organic frameworks (MOFs) constructed with metalloporphyrin units [37,67,68]. Periodic calculations generally consider concerted proton-electron transfer (CPET) as a unified process, as shown in Figure 2. In this case, for example, the reduction of CO_2_ to CO follows the mechanism of the adsorption of CO_2_, the formation of *COOH and *CO, and the desorption of CO. The computational hydrogen electrode (CHE) model [69] was usually used to account for the energy change in adding a proton and an electron, i.e., a hydrogen atom. The effects of pH and applied potential can be incorporated through an energy correction term [70].

## 3. N-Confused Metalloporphyrin

N-confused porphyrins (NCPs) are isomers of porphyrin. The first NCP, 2-aza-21-carbaporphyrin, was synthesized by Latos-Grażyński and Furuta et al. in 1994 [72]. In principle, inverting one, two, or three pyrrole rings of porphyrins could generate a first coordination sphere with N_3_C_1_, N_2_C_2_, or N_1_C_3_ configurations. To date, synthetic efforts reported in the experimental literature have exclusively produced configurations featuring either single or double pyrrole ring inversion, while no successful construction of triply inverted configurations has been achieved. For this reason, theoretical studies mainly focused on N_3_C_1_ and N_2_C_2_ configurations.

**Figure 3:**
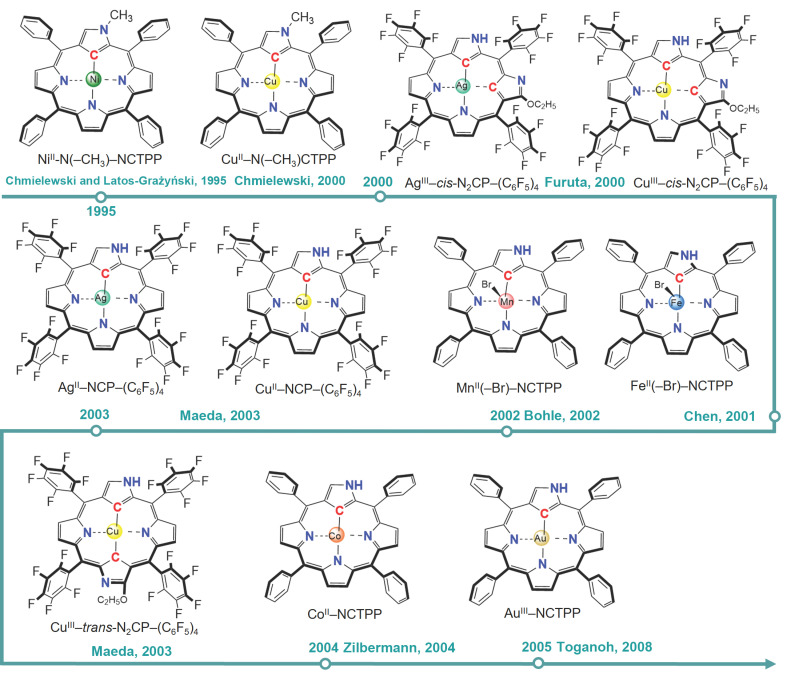
Synthesized N-confused metalloporphyrins to date [73,74,75,76,77,78,83,86,87].

The formal oxidation state of the central metal in a M–N_4_-type metalloporphyrin is +2. Anchoring metal elements into NCPs gives N-confused metalloporphyrins, and the central metal remains a formal oxidation state of +2. The first example of N_3_C_1_-type metalloporphyrins was A Ni(II) complex of 21-(CH_3_)–NCTPP and was reported by Latos-Grażyński et al. in 1995 [73]. Later, in 2000, the same group reported the synthesis of Cu^II^–N(–CH_3_)CTPP [74]. Fe^II^(–Br)–NCTPP (2001) and Mn^II^(–Br)–NCTPP (2002) were synthesized by Hung et al. [75,76]. Cu^II^/Ag^III^/Pd^II^–NCP–(C_6_F_5_)_4_ was synthesized by Furuta et al. in 2003 [77], and Co^II^NCTPP was synthesized by Bettelheim et al. in 2004 [78]. Other than these, N_3_C_1_-type metalloporphyrins with central metals of Mo (2005) [79], Rh (2008) [80], Re (2007) [81], Pt (2000) [82], Au (2005) [83], and Hg (2008) [84] have also been synthesized experimentally. Shao and coworkers (2025) recently reported the synthesis of NiN_3_C- and CoN_3_C-coordinated porphyrin-based COFs [85]. For N_2_C_2_-type metalloporphyrins, the syntheses of complexes with two inverted carbon atoms both in the *ortho*-(*cis*-N_2_CP) and *para*-positions (*trans*-N_2_CP) have been reported. Cu^III^/Ag^III^–*cis*-N_2_CP–(C_6_F_5_)_4_ and Cu^III^–*trans*-N_2_CP–(C_6_F_5_)_4_ were synthesized by Furuta et al. in 2000 and 2003 [86,87], respectively. These N-confused metalloporphyrins are illustrated in Figure 3. Computational studies on these systems with other central metals, such as Co, have been conducted [71].

**Figure 4:**
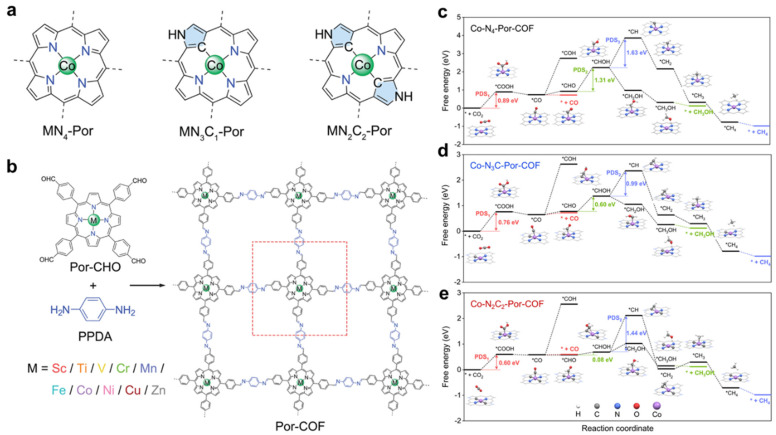
Structural scheme of (**a**) MN_4_-Por, MN_3_C_1_-Por, MN_2_C_2_-Por, and (**b**) Por-COFs. Free energy diagrams of CO_2_RR on (**c**) CoN_4_-Por-COF, (**d**) CoN_3_C1-Por-COF, and (**e**) CoN_2_C_2_-Por-COF. Adapted from reference [71].

The application of N-confused metalloporphyrins as catalysts for CO_2_ reduction has only been demonstrated recently. In 2023, Ren and coworkers reported theoretical studies of N-confused metalloporphyrin-based covalent organic frameworks (Por-COFs) for enhanced electrocatalytic CO_2_RR, where periodic DFT with PBE-D3 functional was used [71]. The study employed a monolayer COF model constructed through imine-linked 5,10,15,20-tetrakis(4-benzaldehyde)porphyrin (Por-CHO) and p-phenylenediamine (PPDA), forming a 2D periodic structure (Figure 4b). Through comprehensive screening of ten 3*d* transition metals (Sc-Zn) based on stability and catalytic selectivity and activity, CoN_4_-Por-COF was identified as a promising candidate for catalyzing CO_2_ reduction to CO (limiting potential *U*_L_ = −0.89 V), while CrN_4_-Por-COF catalyzes CO_2_ reduction to HCOOH (*U*_L_ = −0.78 V). The authors further modified the coordination environment of CoN_4_-Por-COF by inverting one or two pyrrole units to design mono-pyrrole or dipyrrole-inverted N-confused Por-COFs, named CoN_3_C_1_- and CoN_2_C_2_-Por-COFs (Figure 4a). A free energy analysis (Δ*G*) of key intermediates (*COOH vs. *H) revealed the designed N-confused materials effectively suppressed hydrogen evolution (HER) competition. Detailed calculation of the CO_2_ reduction pathway (CO_2_ → COOH → CO → further reduction) showed that the N-confused CoN_3_C_1_- and CoN_2_C_2_-Por-COFs display improved performance in catalyzing CO_2_RR than parent CoN_4_-Por-COF. The limiting potential of reducing CO_2_ to CO decreases from −0.89 V (CoN_4_) to −0.76 V (CoN_3_C_1_) and −0.60 V (CoN_2_C_2_), and applying high potential can yield deep-reduction degree C_1_ products such as CH_3_OH and CH_4_ (Figure 4c–e). Electronic structure analysis revealed that substituting CoN_4_ to CoN_3_C_1_/CoN_2_C_2_ increases the electron density on the Co atom and raises the *d*-band center, thus stabilizing the key intermediates *COOH and lowering the Δ*G* of respective PDSs (PDS refers to potential-determining step). These findings underscore the promise of coordination engineering in COFs, positioning CoN_2_C_2_ and CrN_3_C_1_ as superior candidates for selective CO_2_RR. This theoretical framework not only rationalizes experimental trends but also sets the stage for exploring asymmetric coordination motifs in other transition-metal systems.

Later, Huang and coworkers (2024) [88] synthesized a cobalt porphyrin complex with N-confused coordination, and Shao and coworkers (2025) [85] synthesized a CoN_3_C porphyrin-based COF. Both works demonstrated that the CoN_3_C systems enhance electrocatalytic activity for the reduction of O_2_ to H_2_O. Although the experiments were not for CO_2_RR, the authors confirmed the N_4_ to N_3_C coordination change reduced electron transfer from cobalt to the porphyrin ligand, which is the key for catalytic performance improvements, consistent with the theoretical prediction by Ren’s work [71]. While the periodic DFT approach effectively modeled the electronic structure of monolayer COFs, the study [71] acknowledged limitations including unaccounted interlayer interactions, solvent effects, and kinetic barriers that might lead to overestimated catalytic performance in practical applications. By assuming a CPET process, the kinetic barriers were not estimated in this periodic DFT work.

**Figure 5:**
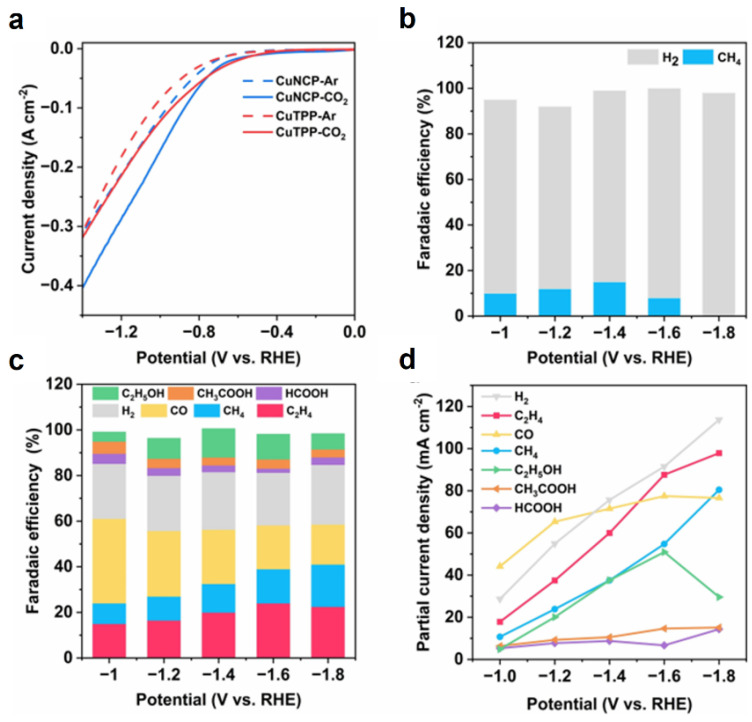
Electrocatalytic CO_2_RR performances of CuTPP and CuNCP under CPE: (**a**) LSVs of CuNCP and CuTPP taken in the flow cell containing 1M KOH purged with Ar or CO_2_. CO_2_RR product distribution and the corresponding FEs at different potentials for (**b**) CuTPP and (**c**) CuNCP. (**d**) Partial current densities of the reduction products at different potentials for CuNCP under CPE [89].

In 2024, Hua and coworkers reported a N-confused copper(II) tetraphenylporphyrin (CuNCP) complex for efficient electrochemical CO_2_ reduction to methane (CH_4_) under pulsed potential electrolysis (PPE) [89]. Two copper porphyrin complexes were synthesized via a modified Geier method: symmetric copper tetraphenylporphyrin (CuTPP, Cu-N_4_ coordination) and asymmetric N-confused copper porphyrin (CuNCP, Cu-N_3_C coordination). Experimental results revealed that CuTPP maintained structural integrity under constant potential electrolysis (CPE) but primarily produced hydrogen (H_2_) (Figure 4b). CuNCP exhibited higher CO_2_ reduction activity (Figure 5a), which is attributed to the asymmetric N_3_C coordination but suffers from structural instability during electrolysis. In situ X-ray absorption spectroscopy (XAS) and transmission electron microscopy (TEM) confirmed the irreversible reduction in CuNCP to Cu^0^ nanoparticles under CPE, leading to diverse products (CO, C_2_H_4_, H_2_, etc.). However, applying PPE (anodic potential: +1.3 V, cathodic potential: −1.6 V, pulse cycle: 5 s/10 s) significantly enhanced CH_4_ selectivity, achieving 60% Faradaic efficiency (FE) with a partial current density of 170 mA cm^−2^ (Figure 5c,d). Combined in situ infrared (IR), Raman spectroscopy, and scanning electrochemical microscopy (SECM) analyses suggested that PPE promotes water dissociation at high anodic bias, consuming OH^−^ and lowering local pH, thereby enriching proton availability for the successive hydrogenation of *CO intermediates and ultimately boosting methane production. Although initial hypotheses proposed dynamic Cu(I)/Cu(II) cycling to restore molecular structure, experimental data ruled out catalyst regeneration, instead highlighting the role of electrolysis protocols in optimizing interfacial chemical environments. This study underscores the trade-off between enhanced catalytic activity and compromised stability in asymmetric coordination (CuN_3_C), and it proposes a novel strategy of tailoring electrolytic conditions to catalyst properties for efficient CO_2_ reduction.

**Figure 6:**
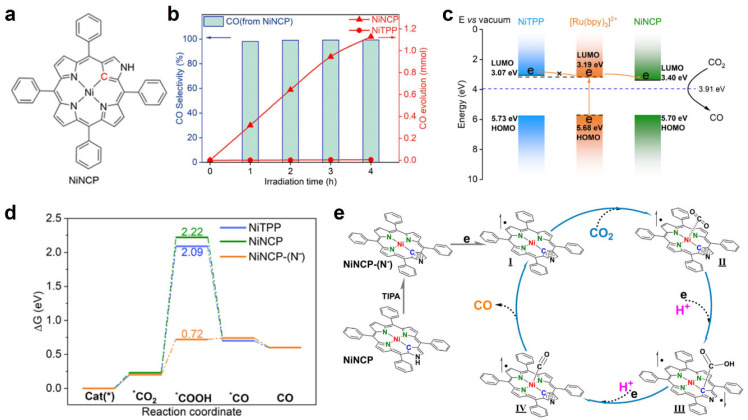
(**a**) Structure of NiNCP. (**b**) CO evolution amount comparison for NiNCP and NiTPP, FE_CO_ for NiNCP. (**c**) Energy level alignment and electron transfer among the [Ru(bpy)_3_]^2+^ photosensitizer, NiTPP and NiNCP cocatalysts, and the CO_2_/CO redox pair. (**d**) Free energy profiles for the CO_2_ reductions with three catalysts. (**e**) Proposed mechanism of the photocatalytic CO_2_RR with NiNCP. Adapted from reference [36].

In 2024, Peng and coworkers reported a nickel(II) N-confused tetraphenylporphyrin complex (NiNCP) (Figure 6a) that efficiently photocatalyzes CO_2_ to CO reduction in acetonitrile solution under irradiation from a blue LED (420 nm), using [Ru(bpy)_3_]^2+^ as a photosensitizer and triisopropanolamine (TIPA) as a sacrificial electron donor [36]. The complex achieved a turnover number (TON) of up to 217,000 with 98% selectivity (Figure 6b). In their experiments, the authors synthesized NiNCP by improving upon the Lindsey and Choi method, confirming its structure through characterization techniques such as MALDI-TOF, XPS, and ^1^H NMR. They discovered that the acidic N–H group in NiNCP is deprotonated by TIPA during the catalytic process, forming an anionic active species, NiNCP^−^. Photocatalytic experiments revealed that NiNCP produced 0.32 mmol of CO within the first hour of the reaction, and the TON increased significantly as the catalyst concentration decreased (Figure 5b). Through steady-state fluorescence and time-resolved spectroscopy analyses, the authors found that NiNCP efficiently extracts electrons from the photosensitizer, with its LUMO energy level (3.40 eV) well matched to those of the photosensitizer (3.19 eV) and the CO_2_/CO couple (3.91 eV) (Figure 6c). Furthermore, DFT calculations indicated that NiNCP^−^ attacks the carbon atom of CO_2_ via a ligand-centered Lewis basic site, forming a stable *COOH intermediate stabilized by a strong C–C bond (1.5 Å), rather than following the traditional metal-centered activation pathway, thereby significantly reducing the reaction energy barrier (Figure 6d,e). The work redefines the role of coordination asymmetry, demonstrating that ligand participation—not just metal tuning—can unlock unprecedented catalytic efficiency.

To our knowledge, only the aforementioned studies have been reported on N-confused porphyrins for CO_2_ research. However, additional works exist regarding metallated NCP complexes applied to the oxygen reduction reaction [85,88]. In the next section, we will discuss the O/S-substituted metalloporphyrins.

## 4. O/S-Substituted Metalloporphyrin

Since one oxygen or sulfur atom is equivalent to one NH group, directly substituting MN_4_ to MN_3_O or MN_3_S of metalloporphyrin leads to the formal oxidation state of the central metal to +1. If one wants to maintain a +2 oxidation state of M, an additional axial ligand, typically a Cl atom, is often introduced. Similarly, when two NH groups are replaced to form N_2_S_2_ or N_2_O_2_-coordinated metalloporphyrins, the central metal is coordinated by two Cl ligands. Experimentally, a few O/S-coordinated metalloporphyrins have been synthesized, including Fe/Co/Ni/Cu/Zn/Mn/Re-21-oxaporphyrin (N_3_O) [90,91,92,93,94], Li/Fe/Ni/Cu/Ru/Rh/Pd/Re/Hg-21-thiaporphyrin (N_3_S) [93,95,96,97,98,99,100], Ni-21,23-dioxaporphyrin (N_2_O_2_) [90], and Ru/Re-21,23-dithiaporphyrin (N_2_S_2_) [101,102]. To date, no experimental studies have reported the successful synthesis of metalloporphyrin complexes incorporating three heteroatoms, such as N_1_O_3_ or N_1_S_3_. Moreover, MOFs or COFs that incorporate metalloporphyrins with oxygen or sulfur have not yet been reported, although their synthesis looks feasible. In principle, oxygen and sulfur can be interchanged and combined to form metalloporphyrin complexes, such as MN_2_OS porphyrins. Such structures have not yet been synthesized experimentally, but they have been studied by computational works [67].

**Figure 7:**
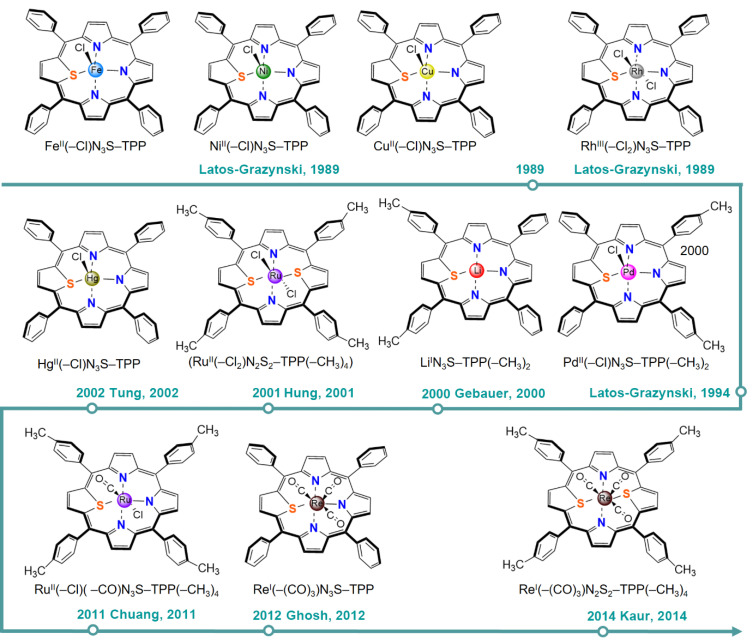
Synthesized S-substituted metalloporphyrins to date [93,95,96,97,98,99,100,101,102].

Latos-Grażyński and co-workers made pioneering efforts in the field of heteroatom-containing porphyrins synthesis. They reported the first synthesis of Cu(II), Fe(II), and Ni(II) complexes of 21-thiaporphyrin (N_3_S–TPP) in 1989 [95]. X-ray diffraction showed that the large radius of the S atom disrupts the planar structure in MN_4_ porphyrins. All these three metalloporphyrins contain an axial chloride ligand, so as to maintain the the metal center in the +II oxidation state. For Ni^II^(–Cl)N_3_S–TPP, Latos-Grażyński and co-workers carried out chemical and electrochemical reduction to prepare its one-electron reduced Ni^I^N_3_S-TPP (1989) [103]. The same group synthesized Rh^III^(–Cl_2_)N_3_S–TPP in 1989 [96] and Pd^II^(–Cl)N_3_S–TPP(–CH_3_)_2_ in 1994 [97]. In 2000, LiN_3_S–TPP(–CH_3_)_2_ was synthesized by Arnold et al. [98]. Hg^II^(–Cl)N_3_S–TPP was prepared by Hwang et al. in 2002 [99]. Later, Ru^II^(–Cl)(–CO)N_3_S–TPP(–CH_3_)_4_ (2011) [100] and Re^I^(–(CO)_3_)N_3_S–TPP (2012) [93] were reported by Hung et al. and Ghosh et al., respectively. For N_2_S_2_-porphyrins, the large atomic radius of S atom disrupts the planar stability, so only N_2_S_2_-porphyrins containing the heavy metal Ru and Re have been synthesized so far, i.e., Ru^II^(–Cl_2_)N_2_S_2_–TPP(–CH_3_)_4_ (2001) [101] and Re^I^(–(CO)_3_)N_2_S_2_–TPP(–CH_3_)_4_ (2014) [102]. These S-substituted metalloporphyrins are illustrated in Figure 7.

**Figure 8:**
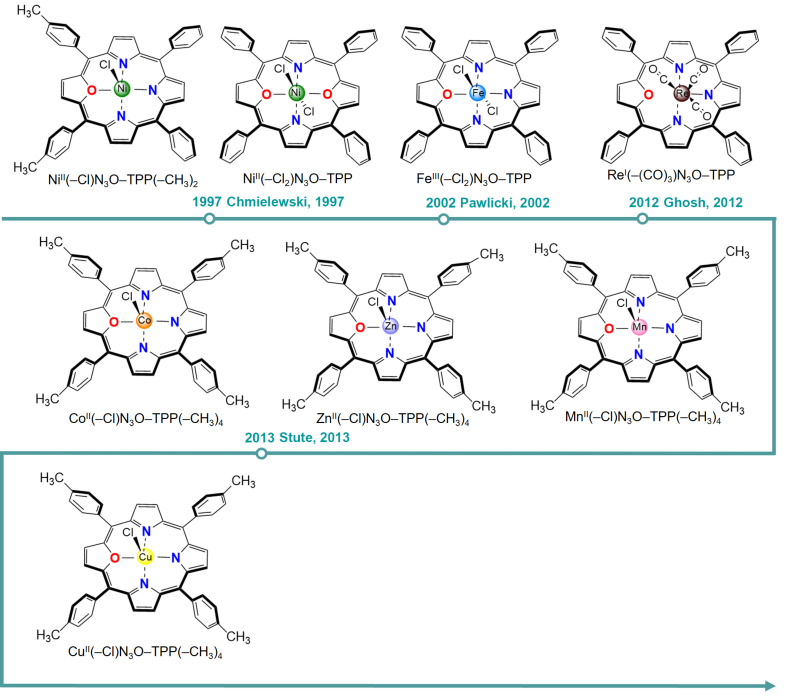
Synthesized O-substituted metalloporphyrins to date [90,91,93,94].

The first example of O-coordinated metalloporphyrins was synthesized in 1997. Latos-Grażyński and co-workers synthesized a five-coordinated Ni(II)-21-oxaporphyrin with an axial chloride ligand, Ni(–Cl)N_3_O–TPP, in which the N_3_O moiety adopts a planar configuration [90]. Subsequently, they conducted an electroreduction experiment to convert it into a four-coordinated Ni^I^N_3_O-TPP. In 2002, the same group also reported the formation of Fe^III^-21-oxaporphyrin [91]. In 2012, Ghosh et al. synthesized Re^I^(–(CO)_3_)N_3_O–TPP [93]. In 2013, Gloe and co-workers reported the Mn(II), Co(II), Zn(II) and Cu(II) complexes of N_3_O porphyrins [94]. For the N_2_O_2_ system, Ni^II^-21,23-dioxaporphyrin was prepared in 1997 [90]. There are two chloride ligands on Ni, and the NiN_2_O_2_ motif attains a planar structure. These O-substituted metalloporphyrins are illustrated in Figure 8. The chemical properties of heteroatom-containing porphyrins and metalloporphyrins have been summarized in several review articles [46,47,48,104,105].

**Figure 9:**
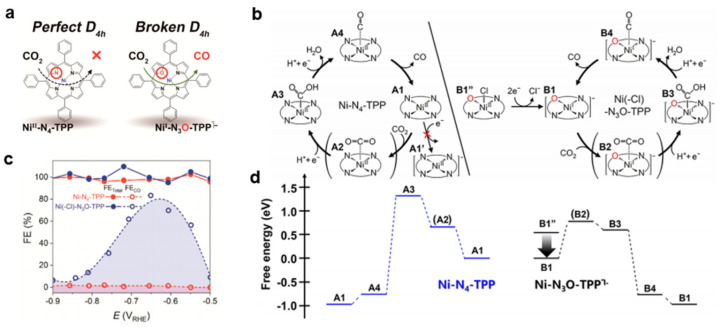
(**a**) Structures of NiN_4_-TPP and NiN_3_O–TPP. (**b**) Suggested CO_2_RR mechanism on NiN_4_–TPP and NiN_3_O–TPP. (**c**) FE_Total_ and FE_CO_ of CO_2_-to-CO conversion in an H-type electrochemical cell. (**d**) DFT-calculated free energy profiles when a potential of −0.6 V vs. RHE is applied. Adapted from reference [27].

Studies concerning the catalytic performances of heteroatom-coordinated metalloporphyrins were only available recently. In 2021, Choi and co-workers investigated the effects of a mono-oxygen substitution in nickel tetraphenylporphyrin (NiN_4_–TPP) on electrocatalytic CO_2_ reduction [27]. The modified nickel-21-oxatetraphenylporphyrin, the Ni(–Cl)N_3_O–TPP catalyst (Figure 9a), was synthesized via a tailored Lindsey method using 2,5-bis (phenylhydroxymethyl) furan as the precursor, followed by metalation and characterization. Electrodes were prepared by mixing the porphyrins with carbon black, and the CO_2_RR was conducted in a 0.5 M KHCO_3_ solution. Ni(–Cl)N_3_O–TPP displays much better electrocatalytic CO_2_RR activity and selectivity than NiN_4_–TPP. NiN_4_–TPP exhibits predominant H_2_ formation over the whole potential range, but minor CO formation is detected at a high overpotential region below −0.75 V_RHE_ with a maximum FE_CO_ of only ca. 2%. (Figure 9c). In sharp contrast, Ni(–Cl)N_3_O–TPP shows an onset potential of CO formation at approximately −0.55 V_RHE_, and its maximum FE_CO_ reaches ca. 80% at −0.65 V_RHE_.

The improved performance was attributed to the broken *D*_4h_ symmetry in Ni(−Cl)N_3_O−TPP, which results in an increase in the Ni redox potential yielding Ni^I^. NiN_4_-TPP shows no reduction between −0.4 and −0.8 V_SHE_, while Ni(–Cl)N_3_O–TPP is reduced at −0.62 V_SHE_. This is also supported by DFT calculations, where their respective calculated reduction potential is −1.04 and −0.54 V_SHE_. As a result, the catalytically active species are different under the applied potential. Ni^II^ in NiN_4_–TPP and Ni^I^ in NiN_3_O–TPP are the actual chemical species responsible for the CO_2_RR. In DFT calculations using the M06 functional, the authors assumed a CPET mechanism under an applied potential of –0.6 V_RHE_, and the reaction follows the steps of CO_2_ adsorption, the formation of *COOH and *CO, and the desorption of CO (Figure 9b). The [NiN_3_O–TPP]^–^ greatly stabilizes the critical species *COOH (Figure 9d), thus leading to a much lower overpotential. In comparison, the calculated COOH binding energy for NiN_4_-TPP is −0.77 eV, and it is −1.73 eV for [Ni–N_3_O–TPP]^–^. The authors pointed out that the weak ligand field formed by N_3_O coordination reduced the energy required for Ni to redistribute electrons to the ligand, thus increasing its Lewis acidity for forming a strong Ni–C bond and stabilizing the *COOH intermediate. At the same time, the lowering of the empty Ni *d* orbital energy due to this electron redistribution results in a positive shift in its reduction potential. Meanwhile, the author also found that the Ni(–Cl)N_3_O–TPP complex suffers from instability over a potential of <−0.6 V_RHE_, posing a challenge for its practical usage. This combined spectroscopic and computational study revealed that the broken ligand-field symmetry is the key for active CO_2_ electrolysis, and controlling the ligand-field strength would be a challenge for future synthesis.

In 2024, He and coworkers [35] reported similar nickel porphyrin catalyst systems in photocatalytic CO_2_ reduction. They incorporated O and S into tetracarboxyphenyl ([–TPP–(COOH)_4_]) nickel porphyrin (NiN_4_-Por) and synthesized Ni(Cl)ON_3_-Por and Ni(Cl)SN_3_-Por. Cyclic voltammetry indicated that the reduction potentials of O/S-substituted species are more positive than the original N_4_-Por. The values are −1.52 V vs. Fc/Fc^+^ for NiN_4_-Por, −1.17 V for Ni(Cl)ON_3_-Por, and −0.82 V for Ni(Cl)SN_3_-Por. DFT calculations at the B3LYP-D3 level showed that the lowest unoccupied molecular orbitals (LUMOs) are lowered from NiN_4_-Por (−2.41 eV) to Ni(Cl)ON_3_Por (−2.87 eV) and Ni(Cl)SN_3_Por (−3.06 eV). The lowering of the LUMO suggests higher electron-accepting capability, and it was used to support the positive shift in reduction potential observed in experiment. Although it is possible to give the calculated reduction potential values, the work did not report them.

**Figure 10:**
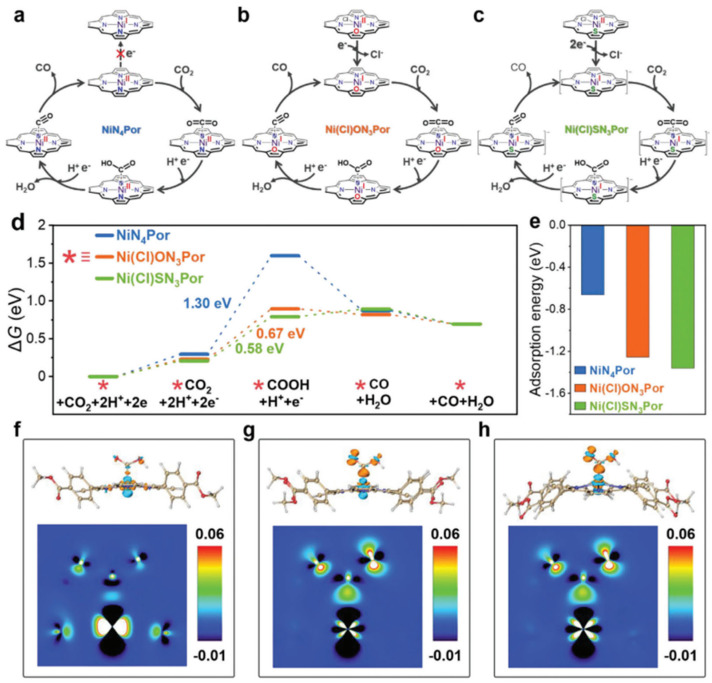
(**a–c**) Schematic illustration of catalytic cycles of NiN_4_–Por, Ni(Cl)ON_3_–Por, and Ni(Cl)SN_3_–Por for CO_2_-to-CO conversion; (**d**) the calculated Gibbs free energy diagrams of CO_2_-to-CO conversion; (**e**) the calculated adsorption energy of *COOH; (**f**–**h**) electron density difference plots of NiN_4_Por, Ni(Cl)ON_3_Por, and Ni(Cl)SN_3_Por in rate-determining step (sky blue and orange indicate electron depletion and accumulation, and the isovalue is 0.01) [35].

Then, the authors conducted photocatalytic experiments with a 465 nm LED lamp in CH_3_CN/H_2_O mixture (*v*/*v*, 4:1) solution and with [Ru(bpy)_3_]Cl_2_ as the photosensitizer and triethanolamine (TEOA) as the electron sacrificial agent. Ni(Cl)ON_3_-Por and Ni(Cl)SN_3_-Por have remarkable CO_2_RR activity, with a CO production rate of 24.7 and 38.8 mmol g^−1^ h^−1^ with a selectivity of 94.0 and 96.4%, respectively (with H_2_ as the by-product), outweighing that of NiN_4_–Por (a CO production rate of 6.6 mmol g^−1^ h^−1^ and selectivity of 82.8%). At the same time, the authors also examined the CO production when using H_2_N_4_–Por, HON_3_–Por, and HSN_3_P–Por as catalysts. They are far less than the CO production from the Ni-porphyrin counterparts, suggesting the Ni center to be the active site. Since the reduction potential of the photosensitizer, [Ru(bpy)_3_]Cl_2_ (PS), PS^+^/PS* is at −1.30 V vs. Fc/Fc^+^, the active catalytic species were proposed to be Ni^II^ in [NiN_4_–Por]^0^, Ni^I^ in [NiON_3_–Por]^0^ (after one-electron reduction), and Ni^I^ in [NiSN_3_Por]^−^ (after two-electron reduction) (Figure 10a–c). Then, these active species followed a CPET mechanism, the PDS was the formation of *COOH, and the energy barrier decreased from 1.30 to 0.67 and 0.58 eV for N_4_ to N_3_O and N_3_S systems (Figure 10d). Similarly, the authors attributed such changes to the disruption of *D*_4h_ symmetry in O/S-substituted Ni-porphyrin. The calculated energy level of Ni *d*_z^2^_ orbital is elevated in the NiON_3_–Por and NiSN_3_–Por (where the writer believed the comparisons should be made between the active species), thus enhancing the adsorption of CO_2_ (Figure 10e). At the same time, the increased electron density on Ni (supposedly, of the active species) stabilizes the *COOH intermediate (Figure 10f–h) and finally leads to faster CO_2_-to-CO conversion. This work demonstrates how O/S-substitution can alter the electronic states of Ni-porphyrin catalysts and improve their catalytic performance.

To our knowledge, experimental studies on O- or S-substituted metalloporphyrins for CO_2_ reduction have been limited to Ni metal centers. Studies that involve other metal centers like Co or Fe are theoretical works.

**Figure 11:**
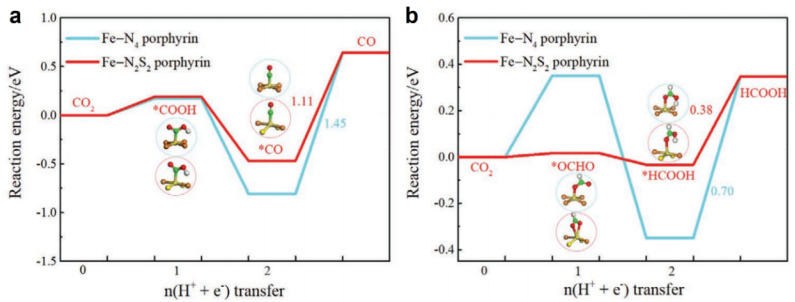
Free energy diagrams of CO_2_RR to (**a**) CO and (**b**) HCOOH and corresponding intermediates on the Fe–N_4_ porphyrin and Fe–N_2_S_2_ porphyrin (at 0 V vs. RHE). The values in the figures refer to the free energy change for the potential-determining step. The asterisks mean intermediates bind at the active site [37].

In 2021, Lu et al. reported a DFT study on Fe-N_2_S_2_ tetraphenylporphyrin (TPP) and predicted that it can improve CO_2_ reduction performance to multiple C1 products, including CO, HCOOH, CH_3_OH, and CH_4_ [37]. The systems are molecular complexes. For the computations, the VASP 6.1.1 software was used with the PBE-D3 functional and implicit solvent model (water as solvent). The computational hydrogen electrode (CHE) model was used. Fe–N_2_S_2_ porphyrin forms a non-coplanar framework with elongated Fe–N bonds (2.14 Å vs. 2.04 Å in Fe–N_4_). The band gap of the Fe–N_2_S_2_ porphyrin (0.34 eV) is much lower than that of the Fe–N_4_ porphyrin (1.62 eV), due to the raised orbital level of *d*-orbitals of Fe and *p*-orbitals of N. The electronic density of states (DOS) reveals additional Fe *d*_z²_ orbital contributions in Fe–N_2_S_2_, promoting intermediate adsorption in the axial direction. The Fe atom in Fe–N_2_S_2_ was found to have a higher electron density than the Fe atom in Fe–N_4_ porphyrin. Fe–N_2_S_2_ exhibits slightly stronger CO_2_ adsorption (*E*_ads_ = –0.20 eV) compared to Fe–N_4_ (*E*_ads_ = –0.16 eV), attributed to increased electron transfer from Fe to CO_2_. Note that the adsorption energy difference is about 0.04 eV or 0.9 kcal/mol, which is within the accuracy of DFT calculations. For the CO_2_-to-CO pathway, the PDS of both Fe–N_4_ and Fe–N_2_S_2_ systems is the desorption of CO, with respective Δ*G* of 1.45 eV and 1.11 eV (Figure 11a). For the HCOOH pathway, the PDS is the desorption of HCOOH for both FeN_4_ and FeN_2_S_2_ porphyrins and the respective Δ*G* of 0.70 eV and 0.38 eV (Figure 11b). For the CH_3_OH pathway, the PDS is CH_3_OH desorption for the Fe–N_4_ porphyrin and *CO→*CHO step for the Fe–N_2_S_2_ porphyrin, with the respective Δ*G* of 0.90 eV and 0.40 eV. For the CH_4_ pathway, the PDS is the *CO→*CHO step for the Fe–N_4_ porphyrin, and *CH_3_OH→*CH_3_ step for FeN_2_S_2_ porphyrin, with the respective Δ*G* of 0.73 eV and 0.56 eV. As a result, the calculated limiting potentials *U*_L_ for all pathways were lowered in FeN_2_S_2_ in comparison to the Fe–N_4_ case. The respective values are listed in Table 1.

On the Fe–N_4_ porphyrin, *U*_L_ follows the sequence HCOOH > CH_4_ > CH_3_OH > CO. On the Fe-N_2_S_2_ porphyrin, *U*_L_ follows the sequence HCOOH > CH_3_OH > CH_4_ > CO, with the value of −0.38 V for HCOOH and −0.40 V for CH_3_OH, surpassing most reported catalysts. At the same time, since their values of *U*_L_ are very close, the HCOOH and CH_3_OH product channels are in great competition. Compared to conventional Fe–N_4_, Fe–N_2_S_2_ porphyrin shifts product selectivity toward HCOOH and CH_3_OH, with reduced energy barriers for key steps such as *CO → *CHO. An important drawback of the Fe–N_2_S_2_-system is that the hydrogen evolution also becomes easier. The calculated free energy change of H^+^ + e^−^ → *H → H is 0.59 eV on the Fe–N_4_ porphyrin and 0.26 on the Fe–N_2_S_2_ porphyrin. This work underscores the potential of double-heteroatom coordination strategies in designing high-efficiency electrocatalysts.

**Figure 12:**
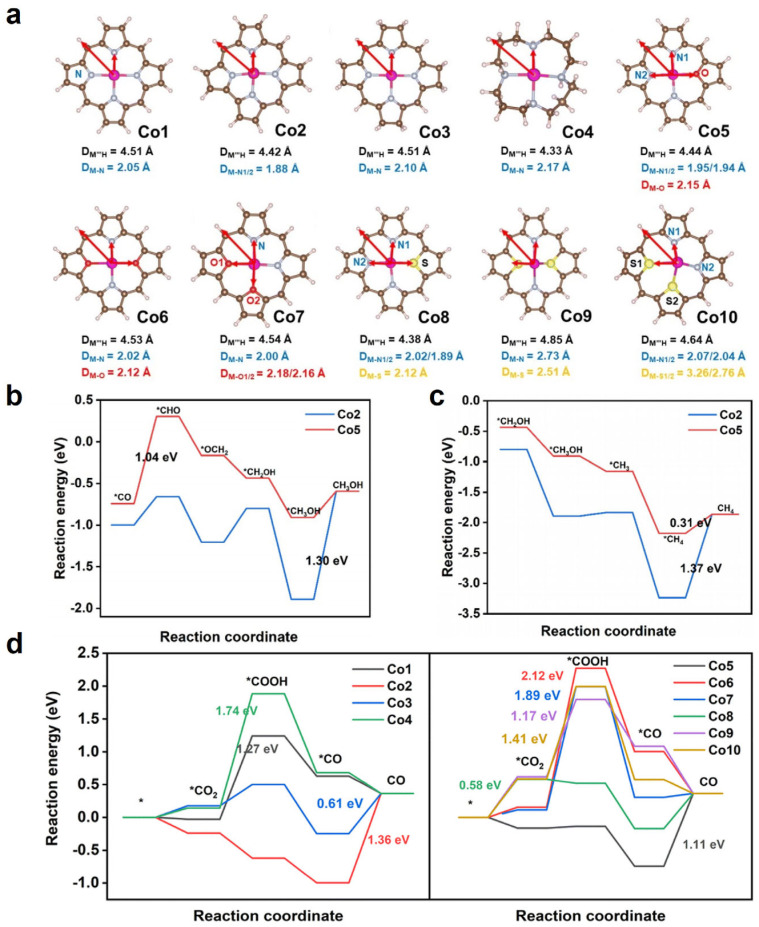
(**a**) Structures of Co–X (X = N, O, S). Free energy diagrams of CO_2_ reduction pathway to (**b**) CH_3_OH and (**c**) CH_4_ on Co5. (**d**) Free energy diagrams of CO_2_RR to CO on Co-X complexes. Adapted from reference [106].

In 2023, Qi and co-workers [106] reported a theoretical study investigating a series of O- or S-substituted cobalt porphyrin molecular complexes (Figure 12a, where the peripheral ligands are H atoms) for electrochemical CO_2_RR. The authors performed DFT calculations using Gaussian 09 with a ωB97XD-D3BJ functional and SMD implicit solvent model with water as the solvent. The computational hydrogen electrode model was used. Based on the Co–N_4_ porphyrin (Co1), they modified the ring structure and designed three structures, including cobalt corrole (Co2), cobalt octahydroporphyrin (Co3), and cobalt 1,5,9,13-tetraazacyclohexadecane (Co4). In terms of the core modification, they introduced O and S heteroatoms and formed six structures, including cobalt 21-oxaporphyrin (Co5, N_3_O), cobalt 21,23-dioxaporphyrin (Co6, N_2_O_2_), 21,22-dioxaporphyrin (Co7, N_2_O_2_), cobalt 21-thiaporphyrin (Co8, N_3_S), cobalt 21,23-dithiaporphyrin (Co9, N_2_S_2_), and cobalt 21,22-dithiaporphyrin (Co10, N_2_S_2_). Here, we focus on the comparison between Co5–Co10 and Co1. The authors considered different products of CO_2_ reduction, including CO, CH_3_OH, and CH_4_. The product channel of HCOOH and the HER was not discussed in this work.

Similarly, the core-modification approach decreased the band gap as compared to the CoN_4_ porphyrin. This serves as a hint that the intramolecular electron transfer of these cobalt catalysts would be improved during the CO_2_RR process. The PDS of CO_2_-to-CO reduction on Co1 (N_4_), Co6 (N_2_O_2_), Co7 (N_2_O_2_), Co9 (N_2_S_2_), and Co10 (N_2_S_2_) is the formation of *COOH, where their respective Δ*G* values are 1.27, 2.12, 1.89, 1.17, and 1.41 eV. The PDS of Co5 (N_3_O) is CO desorption with Δ*G* of 1.11 eV, indicating a promising catalyst for the further hydrogenation reactions of CO into CH_3_OH (*U*_L_ = −1.04 V) and CH_4_ (*U*_L_ = −1.04 V) (Figure 12b,c). The PDS of Co8 (N_3_S) is CO_2_ adsorption, with a minimum Δ*G* of 0.58 eV among the studied systems, indicating the best catalytic activity for CO_2_-to-CO reduction (Figure 12d). Co8 also has the smallest band gaps (3.40 eV), highlighting its strongest reduction capability. The authors concluded that single O/S-substituted cobalt porphyrin exhibits higher activity than double O/S-substituted cobalt porphyrin, and CoN_3_S is better than CoN_3_O. The adsorption energies of CO in the designed complexes were analyzed. Change analyses showed that Co5–Co10 transferred more electrons (−0.046 |e| ~ −0.182 |e|) to CO, in comparison to Co1 (0.149 |e|, indicating electron transfer from CO to Co1), hence, a stronger CO binding, and this facilitates the formation of deeper reduction products like CH_3_OH and CH_4_. They also claimed that the broken D_4_h symmetry altered the electron density of the Co porphyrin and improved CO_2_RR performance.

**Figure 13:**
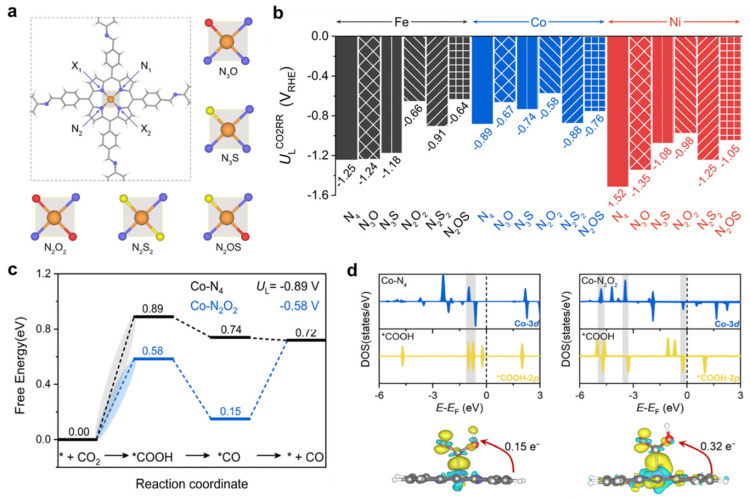
(**a**) Unit cell of the M–N_4_-Por-COF (M = Fe/Co/Ni) and the constructed N_x_O_y_S_z_ coordination models. (**b**) Limiting potential of CO_2_RR-to-CO of Fe/Co/Ni–N_x_O_y_S_z_-Por-COFs. (**c**) Free energy diagrams of CO_2_RR on Co–N_4_-and Co–N_2_O_2_-Por-COFs. (**d**) Projected electronic densities of states (PDOS) and charge density difference (CDD) of *COOH intermediates adsorbed on Co–N_4_ and Co–N_2_O_2_-Por-COFs. Adapted from reference [67].

In 2024, Ren et al. [67] performed periodic DFT calculations with PBE-D3 functional on O/S-substituted metalloporphyrin-based COFs (Por-COFs) for electrocatalytic CO_2_ reduction. This work designed 15 types of heteroatomic Por-COFs, featuring M–N_x_O_y_S_z_ (M = Fe, Co, Ni; x + y + z = 4) centers, including single-substituted models: N_3_O, N_3_S, and double-substituted models: N_2_O_2_, N_2_S_2_, and N_2_OS (Figure 13a). It focused on the electrocatalytic reduction of CO_2_ to CO and assumed a CPET mechanism. The PDS for all the target M–N_x_O_y_S_z_-Por-COFs is the formation of the *COOH intermediate. Figure 13b summarizes the corresponding theoretical limiting potentials *U*_L_. In general, changing the coordination from N_4_ to N_x_O_y_S_z_ shifts the *U*_L_^CO_2_RR^ towards the positive direction, meaning an easier reduction and thus higher CO_2_-to-CO catalytic activity. The majority of the designed M–N_x_O_y_S_z_-Por-COFs display a distinct preference for CO_2_RR. Exceptions are Ni–N_3_O, Co–N_3_O, Co–N_3_S, and Co–N_2_S_2_ systems, which show a slight preference for HER. Based on calculated *U*_L_ values, the four CO_2_-to-CO catalyst candidates ranked at the top are Co–N_2_O_2_, Fe–N_2_OS, Fe–N_2_O_2_, and Co–N_3_O-Por-COFs. The best designed system, Co–N_2_O_2_-Por-COF, achieved a limiting potential (*U*_L_^CO_2_RR^) of −0.58 V, outperforming the parent Co–N_4_-Por-COF (*U*_L_^CO_2_RR^ = −0.89 V) (Figure 13c). Electronic structure analysis attributed this improvement to enhanced orbital overlap between Co 3*d* orbitals and *COOH 2*p* orbitals, alongside increased charge transfer (0.32 e^−^ in Co–N_2_O_2_ vs. 0.15 e^−^ in Co–N_4_), stabilizing the critical *COOH intermediate (Figure 13d). In comparison, the *U*_L_ values of the Ni-based systems are more negative than the Fe- and Co-based catalysts. Ren et al. predicted that the *U*_L_ follows the order of Ni–N_4_ (−1.52 V) < Ni–N_3_O (−1.35 V) < Ni–N_3_S (−1.08 V). Although the Ni–N_3_S and Ni–N_3_O systems were not the best, this prediction aligns with the experimental superiority of Ni(Cl)–SN_3_Por and Ni(Cl)ON_3_Por observed in the photocatalytic CO_2_ reduction [35], affirming the accuracy of this computational work. Furthermore, the machine learning model (Gradient Boosting Regression, GBR) further identified central metal mass (*m*) and metal-ligand bond lengths (*d*_M-X_) as pivotal descriptors, underscoring the synergy between metal identity and coordination environment. This work not only predicts high-performance Por-COF candidates but also establishes coordination engineering as a universal strategy for tailoring COF-based electrocatalysts.

The three computational studies mentioned above, although they involve different peripheral ligands, in part share the same first coordination spheres. All calculations assumed a CPET mechanism, and the computational results showed both similarities and differences. The similarities include that, after O/S coordination, the band gap of the metal porphyrin decreases, the electron density at the metal center increases, and the adsorption energies of key intermediates (such as CO_2_, CO, COOH, and others) change, leading to different catalytic activities for CO_2_ reduction.

As for the differences, we highlight the following for instance. For the FeN_2_S_2_ system, both Lu et al. and Ren et al. predicted a lower *U*_L_ for CO_2_-to-CO conversion compared to the FeN_4_ system (Table 1). However, the former work (employed cluster model) identified CO desorption as the PDS, whereas the latter work (employed periodic model) identified the COOH formation as the PDS. For the Co–N_x_O_y_ system, Qi et al. (employed cluster model) suggested that the single-substituted Co–N_3_O-Por performs better than the double-substituted Co–N_2_O_2_-Por system, and, in comparison to Co–N_x_S_y_ systems, S-substitution is better than O-substitution. However, Ren et al.’s calculations (employed periodic model) yielded the opposite conclusion, favoring the double-substituted Co–N_2_O_2_-Por-COF system, and O-substitution is better than S-substitution for Co–N_4_-system. These discrepancies may arise from ligand effects and, of course, the differences in computational models and methods.

Theoretical studies by Ren et al. in 2023 [71] and 2024 [67] employed the same Por-COF model and DFT method, allowing us to compare the calculated CO_2_-to-CO performance of N-confused and O/S-substituted Co-Por-COF systems. These systems shared the same PDS, i.e., the formation of the *COOH intermediate. As shown in Table 1, for singly substituted Co-Por-COF, the order of calculated *U*_L_ (thus the order of catalytic performance) is CoN_3_O (−0.67 V) > CoN_3_S (−0.74 V) > CoN_3_C (−0.76 V) > CoN_4_ (−1.27 V); for double-substitution, the order of *U*_L_ is CoN_2_O_2_ (−0.58 V) > CoN_2_C_2_ (−0.60 V) > CoN_2_S_2_ (−0.88 V). Based on these *U*_L_ values, all these systems improved the catalytic performance as compared to the original CoN_4_ system, because O, S, and C substitutions increased the electron density of the Co center, thereby strengthening the binding with *COOH. CoN_2_O_2_ is the best, and double substitution is better than single substitution for C and O, but the trend reverses for S. This latter decline was attributed to the fact that the large atomic radius of S distorts the catalyst structure, reducing orbital overlap and weakening binding to *COOH. This underscores the necessity of balancing electronic modulation and structural stability in heteroatom-substitution strategies, providing crucial guidance for the rational design of high-performance CO_2_ reduction catalysts.

## 5. Summary and Outlook

In this review, we have summarized the recent progress in the application of N-confused and O/S-substituted metalloporphyrins-based systems for CO_2_ reduction, both experimentally and theoretically. In general, the first coordination sphere modification by C, O, and S atoms represents an effective method to improve the catalytic activity and selectivity of CO_2_ reduction. By breaking the symmetry of traditional M–N_4_ coordination, these heteroatoms induce distinct electronic perturbations that optimize intermediate adsorption and lower reaction energy barriers. Furthermore, these heteroatom coordination structures significantly influence product selectivity. The catalytic performances of these systems are summarized in Table 2, where properties including reduction potential, products and yields, and Faradaic efficiencies are present. It shows that the reactivity changes depend on the type of substituted atoms, the metal center, and experimental conditions. In addition, computational models and methods also affect the results.

Experimentally, a Cu–NCP complex was found to improve the electroreduction of CO_2_-to-CH_4_ products under pulsed potential electrolysis (PPE), mainly because PPE lowered the local pH. A Ni–NCP complex was reported to improve the photoreduction of CO_2_-to-CO, and it was attributed to a ligand-involved mechanism. In addition, Ni–N_3_(Cl)O- and/or Ni–N_3_(Cl)S-porphyrin systems were found to improve the activity and selectivity of CO_2_-to-CO electrochemically and photochemically. The detailed underlying mechanisms of enhanced catalytic reactivity are different for C and O/S substitution, as well as for Cu and Ni. For the Ni-porphyrin system, common findings include that the C/O/S substitutions shift the reduction potential to positive, the Ni^I^ species are the active catalytic species, and S substitution is better than O substitution.

Both electrocatalytic works mentioned that the modified porphyrin systems experienced critical instability issues, indicating that the asymmetric coordination may weaken macrocyclic stability under redox cycling. Under high potential, Cu-NCP electrolysis to Cu clusters/nanoparticles, applying PPE only delayed and cannot avoid the decomplexation of Cu-NCP [89]. The Ni ions were dissolved from the Ni(–Cl)N_3_O-porphyrin system to form metallic Ni or oxidized NiO and Ni(OH)_2_ precipitations [27]. In periodic DFT calculations, the stability of original catalysts is often predicted by computing the formation energy and dissolution potential (*U*_diss_) of the metalloporphyrin materials. Predicting the stability of catalysts during the catalytic process, i.e., under applied potential, is normally beyond the calculation capability, due to the difficulty in modeling the real working conditions. To account for the role of solvent in affecting the stability of the catalysts, one may include explicit solvent molecules, but the computational cost is much higher. Moreover, including the effect of potential during the simulations is computationally challenging.

In contrast, both photocatalytic works reported that the catalysts are stable under the experimental conditions. It seems to indicate that, in terms of stability, the first coordination modified porphyrins are suitable for photocatalysis reactions, rather than electrocatalytic reactions.

Computational works extended the systems from single substitution to double and mixed substitutions. Most works focus on Fe, Co, and Ni metal centers. Although the computational models and methods are not the same in those works, the calculation results revealed a number of universal observations, no matter whether the substituted atoms are C, O, or S. The heteroatom substitution changes (mainly increases) the electron density of the center metals, which may enhance the electron transfer from M to other incoming species. It decreases the HOMO-LUMO gap (or band gap) of the porphyrin systems, either by lowering the LUMO or raising the HOMO. The energy level of the *d*_z^2^_ orbital was raised; this can enhance the interaction with incoming species with a proper orbital orientation. These electron redistributions and the change in orbital levels can lead to a shift in the reduction potential and stabilization of key intermediates (such as CO_2_, COOH, CO, etc.) during CO_2_RR processes and then change the reaction barrier. For different metals, heteroatom substitution affects the intermediates differently, so the potential determining steps, and thus the selectivity, may differ. The major CO_2_ reduction product of metalloporphyrin complexes is CO and H_2_. After introducing heteroatoms in the first coordination sphere, calculations suggest that Ni systems still prefer CO, Fe systems (like Fe–N_2_S_2_) may prefer HCOOH and CH_3_OH, and Co systems (like Co–N_3_C_1_, Co–N_2_C_2_) have increased preference for CH_3_OH and CH_4_.

Theoretical calculations predicted improved CO_2_RR activity for Fe- and Co-centered porphyrin with C or O or S coordination, and these predictions have not been validated by experiments yet, which suggests a potential avenue for further experimental exploration. We would like to point out that the calculations mentioned above are based on the thermodynamics results as computed from the DFT method by assuming CPET, and the CHE model was usually used. We believe many important issues are worth further exploring. For example, treat the protonation and electron-transfer steps separately [60,61,62,66,107], examine the transition states (as the system may be kinetically controlled) [64], track the flow of electrons to check the oxidation state and clarify the active catalytic species, consider the mechanism that ligands serve as active sites, account for the solvent effect by including explicit solvent molecules, etc. It is worth mentioning that these calculations did not consider factors like catalyst degradation or mass transport in real-world electrolysis. A direct comparison between the overpotential measured in the experiment and the limiting potentials (*U*_L_) calculated by DFT is not anticipated. Nevertheless, comparing the calculated parameters, including *U*_L_ values and activation energies, across different catalysts provides valuable information on the change in catalytic activity and selectivity.

One may be interested in comparing the influence of the first coordination and outer-sphere modification on the catalytic performance of CO_2_ reduction. Here, we take the comparison between NiN_4_–TPP, NiN_3_O-TPP, and Ni–TPP–NI*t*Bu (Ni-1,3-ditert-butylimidazolin-2-ylidenamino-tetraphenylporphyrin) for illustration. Experimentally, the unmodified Ni–N_4_–TPP exhibits extremely low catalytic activity in CO_2_ electroreduction. Through the first coordination sphere modification, the O-coordinated Ni–N_3_O–TPP achieved an FE_CO_ of 80% at −0.65 V_RHE_ and experienced a positive shift in reduction potential [27]. Through outer-sphere modification, adding the NI*t*Bu group to peripheral benzene ring forms Ni–TPP–NI*t*Bu. It increased the FE_CO_ to 62% at −1.10 V_RHE_ but had no obvious effect on the reduction potential [64]. Both strategies improved the CO_2_-to-CO reduction performance, but the underlying mechanisms are not the same. For the former, O-substitution disrupts the *D*_4h_ symmetry of NiN_4_ and redistributes the electron density on Ni. This promotes the formation of the active species Ni^I^ and stabilizes the *COOH intermediate, thus enhancing the CO_2_ to CO reduction. For the latter, appending the NI*t*Bu group on the outer-sphere increases the electron density at the Ni center, for NI*t*Bu is a strong electron-donating group. This facilitates the binding of CO_2_ and improves catalytic performance. We can see that the first coordination sphere affects the electron density of the central metal through ligand-field regulation, whereas the outer-coordination sphere affects it via longer-distance electronic interactions.

In comparison, O-substitution is more effective than appending peripheral NI*t*Bu groups in increasing the catalytic reactivity towards CO_2_-to-CO reduction. However, based on this single comparison, we cannot draw a general conclusion that first coordination modification is more effective than outer-sphere modification. Electron-withdrawing or cationic groups were found to have distinct effects as compared to electron-donating groups. The effects of different substituents were studied for Fe and Co porphyrin systems but not for Ni porphyrin systems [33,34]. We anticipate that combining these two strategies may achieve the maximum enhancement of catalytic performance. In summary, heteroatom-containing metalloporphyrins represent a versatile platform for catalysis for CO_2_ and other small molecules. This approach not only provides a deeper understanding of the structure and activity relationships governing reduction reactions but also offers a clear pathway for the rational design of next-generation electrocatalysts. We propose the following promising directions for future investigations: the synergistic effect between the modification of first- and peripheral coordination sphere modification [108], the incorporation of multi-metal centers within expanded porphyrin analogs [109,110,111], and the integration with composite materials such as carbon nanostructures [112,113], covalent organic frameworks [114], metal–organic frameworks [115,116], or conductive polymers [117,118]. Furthermore, to establish catalyst design principles, mechanistic investigations require not only theoretical and computational approaches but also the experimental monitoring of dynamic processes. Operando spectroscopy (e.g., XAS, SEIRAS, DEMS) has been applied to study the catalytic mechanism of CO_2_ reduction [118,119]. These techniques can capture the reaction intermediates and obtain critical information such as reaction rates, product formation kinetics, and the oxidation states of active sites on catalysts, thereby bridging the gap with theoretical calculations and facilitating better catalyst design.

## Figures and Tables

**Table 1:** Calculated limiting potential (*U*_L_/V) of the products for the H_2_, CO, HCOOH, CH_3_OH and CH_4_ production on metalloporphyrin systems and DFT methods.

	H_2_	CO	HCOOH	CH_3_OH	CH_4_	DFT Method	Ref.
FeN_4_-TPP-Por	−0.59	−1.45	−0.70	−0.90	−0.73	Cluster PBE-D3	[37]
FeN_2_S_2_-TPP-Por	−0.26	−1.11	−0.38	−0.40	−0.56		
FeN_4_-Por-COF	−1.25	−1.25	/	/	/		
FeN_3_S-Por-COF	−1.25	−1.18	/	/	/	Periodic PBE-D3	
FeN_2_S_2_-Por-COF	−1.03	−0.91	/	/	/		[67]
FeN_3_O-Por-COF	−1.50	−1.24	/	/	/		
FeN_2_O_2_-Por-COF	−1.26	−0.66	/	/	/		
CoN_4_-Por	/	−1.27	/	−0.80	−0.80		[106]
CoN_3_S-Por	/	−0.58	/	−0.91	−0.91	Cluster ωB97XD-D3BJ	
CoN_2_S_2_-Por	/	−1.17	/	−0.66	−1.20		
CoN_3_O-Por	/	−1.11	/	−1.04	−1.04		
CoN_2_O_2_-Por	/	−2.12	/	−0.96	−1.40		
CoN_4_-Por-COF	−0.91	−0.89	−1.36	−1.31	−1.63		[71]
CoN_3_C_1_-Por-COF	−0.79	−0.76	/	−0.76	−0.99	Periodic PBE-D3	
CoN_2_C_2_-Por-COF	−0.63	−0.60	/	−0.60	−1.44		
CoN_3_S-Por-COF	−0.75	−0.74	/	/	/	Periodic PBE-D3	
CoN_2_S_2_-Por-COF	−0.84	−0.88	/	/	/		[67]
CoN_3_O-Por-COF	−0.64	−0.67	/	/	/		
CoN_2_O_2_-Por-COF	−0.79	−0.58	/	/	/		
NiN_4_-Por-COF	−1.52	−1.52	/	/	/		
NiN_3_S-Por-COF	−1.16	−1.08	/	/	/		
NiN_2_S_2_-Por-COF	−1.28	−1.25	/	/	/	Periodic PBE-D3	[67]
NiN_3_O-Por-COF	−0.97	−1.35	/	/	/		
NiN_2_O_2_-Por-COF	−1.23	−0.98	/	/	/		

**Table 2:** Summary of different catalyst systems and catalytic performance of metalloporphyrin complexes in CO_2_RR (*E*_red_: reduction potential; *U*_L_: limiting potential; EC: electrocatalysis; PC: photocatalysis; Expt.: experimental; TPP: tetraphenylporphyrin; and Por: porphyrin).

Catalysts	Cores	Product, FE	Potential	Method	Cat. Type	Solvent	Ref.
Iron-TPP	FeN_4_	HCOOH	−0.70 V_RHE_ (*U*_L_)	DFT	EC	H_2_O	[37]
Iron-21,23-dithia-TPP	FeN_2_S_2_	HCOOH CH_3_OH	−0.38 V_RHE_ (*U*_L_) −0.40 V_RHE_ (*U*_L_)				
Cobalt-TPP	CoN_4_	CO, 95%	−0.60 V_RHE_ (*E*_red_)	Expt.	EC	0.5 M NaHCO_3_	[34]
Cobalt 21-thia-Por	CoN_3_S	CO	−0.58 V_RHE_ (*U*_L_)	DFT	EC	H_2_O	[106]
Cobalt 21,23-dithia-Por	CoN_2_S_2_	CH_3_OH	−0.66 V_RHE_ (*U*_L_)				
Cobalt 21-oxa-Por	CoN_3_O	CH_3_OH, CH_4_	−1.04 V_RHE_ (*U*_L_)				
Cobalt 21,23-dioxa-Por	CoN_2_O_2_	CH_3_OH	−0.96 V_RHE_ (*U*_L_)				
CoN_4_-Por-COFs	CoN_4_	CO	−0.89 V_RHE_ (*U*_L_)	DFT	EC	H_2_O	[71]
CoN_3_C-Por-COFs	CoN_3_C	CO, CH_3_OH	−0.76 V_RHE_ (*U*_L_)				
CoN_2_C_2_-Por-COFs	CoN_2_C_2_	CO, CH_3_OH	−0.60 V_RHE_ (*U*_L_)				
Nickel-TPP	NiN_4_	CO, 2% CO, 29%	−0.75 V_RHE_ (*E*_red_) −1.10 V_RHE_ (*E*_red_)	Expt.+DFT Expt.	EC	0.5 M KHCO_3_ DMF	[27] [19]
Nickel-21-oxa-TPP	NiN_3_O	CO, 80%	−0.65 V_RHE_ (*E*_red_)	Expt.+DFT	EC	0.5 M KHCO_3_	[27]
Nickel-TPP(-COOH)_4_	NiN_4_	CO, 82.8%	−1.52 V_Fc+/Fc_(*E*_red_)/−1.29 V_RHE_	Expt.+DFT	PC	CH_3_CN/H_2_O	[35]
Nickel-21-oxa-TPP(-COOH)_4_	NiN_3_O	CO, 94.0%	−1.41 V_Fc+/Fc_(*E*_red_)/−1.18 V_RHE_				
Nickel-21-thia-TPP(-COOH)_4_	NiN_3_S	CO, 96.4%	−0.82 V_Fc+/Fc_(*E*_red_)/−0.60 V_RHE_				
Nickel-TPP	NiN_4_	CO, <5%	/	Expt.+DFT	PC	CH_3_CN	[36]
Nickel-NCTPP	NiN_3_C	CO, 98%	−1.41 V_Ag/Ag+_(*E*_red_)/−1.26 V_RHE_				
Copper-TPP	CuN_4_	CO, <15%	−1.8 V_RHE_(*E*_red_)	Expt.	EC	1 M KOH	[89]
Copper-NCTPP	CuN_3_C	CH_4_, >60%	−1.6 V_RHE_(*E*_red_)				

## Data Availability

Data sharing is not applicable.
